# Novel Nanohybrids Based on Supramolecular Assemblies of Meso-tetrakis-(4-sulfonatophenyl) Porphyrin J-aggregates and Amine-Functionalized Carbon Nanotubes

**DOI:** 10.3390/nano10040669

**Published:** 2020-04-02

**Authors:** Mariachiara Trapani, Antonino Mazzaglia, Anna Piperno, Annalaura Cordaro, Roberto Zagami, Maria Angela Castriciano, Andrea Romeo, Luigi Monsù Scolaro

**Affiliations:** 1 CNR-ISMN, Istituto per lo Studio dei Materiali Nanostrutturati c/o Dipartimento di Scienze Chimiche, Biologiche, Farmaceutiche ed Ambientali, Università di Messina, V. le F. Stagno D’Alcontres 31, 98166 Messina, Italy; mariachiara.trapani@cnr.it (M.T.); annalaura.cordaro@ismn.cnr.it (A.C.); roberto.zagami@ismn.cnr.it (R.Z.); anromeo@unime.it (A.R.); lmonsu@unime.it (L.M.S.); 2 Dipartimento di Scienze Chimiche, Biologiche, Farmaceutiche ed Ambientali, Università di Messina, V. le F. Stagno D’Alcontres 31, 98166 Messina, Italy; apiperno@unime.it; 3 Consorzio Interuniversitario Nazionale di Ricerca in Metodologie e Processi Innovativi di Sintesi, C.I.N.M.P.I.S., Unità Operativa dell’Università di Messina, V. le F. Stagno D’Alcontres, 3198166 Messina, Italy; 4 Consorzio Interuniversitario di Ricerca in Chimica dei Metalli nei Sistemi Biologici, C.I.R.C.M.S.B, Unità Operativa dell’Università di Messina, V. le F. Stagno D’Alcontres, 31, 98166 Messina, Italy

**Keywords:** porphyrin, J-aggregates, carbon nanotubes, nanohybrids

## Abstract

The ability of multiwalled carbon nanotubes (MWCNTs) covalently functionalized with polyamine chains of different length (ethylenediamine, EDA and tetraethylenepentamine, EPA) to induce the J-aggregation of meso-tetrakis(4-sulfonatophenyl)porphyrin (TPPS) was investigated in different experimental conditions. Under mild acidic conditions, protonated amino groups allow for the assembly by electrostatic interaction with the diacid form of TPPS, leading to hybrid nanomaterials. The presence of only one pendant amino group for a chain in EDA does not lead to any aggregation, whereas EPA (with four amine groups for chain) is effective in inducing J-aggregation using different mixing protocols. These nanohybrids have been characterized through UV/Vis extinction, fluorescence emission, resonance light scattering and circular dichroism spectroscopy. Their morphology and chemical composition have been elucidated through transmission electron microscopy (TEM) and scanning transmission electron microscopy (STEM). TEM and STEM analysis evidence single or bundles of MWCNTs in contact with TPPS J-aggregates nanotubes. The nanohybrids are quite stable for days, even in aqueous solutions mimicking physiological medium (NaCl 0.15 M). This property, together with their peculiar optical features in the therapeutic window of visible spectrum, make them potentially useful for biomedical applications.

## 1. Introduction

Carbon nanotubes (CNTs) are intriguing materials with applications ranging from nanotechnology-related devices (i.e., in electronics, energy storage, water treatment, as sensor/biosensor) [1] to drug/probes delivery systems for therapy and diagnosis [2,3].

Functionalized CNTs are widely used to reduce the intrinsic toxicity of “as produced” (pristine) CNTs by increasing the tolerability and the biodegradability in vivo [4,5]. Furthermore, opportunely modified and sized multiwalled CNTs (MWCNTs) are not retained in the organs and can be easily cleared by body excretion [6].

Functional nanomaterials based on CNTs were designed as therapeutic enhancers by combining CNTs with different systems, such as cyclodextrins, biomolecules or porphyrinoids [3]. Recently, some of us reported antiviral- and plasmid/delivery systems [7,8] based on properly functionalized MWCNTs, investigating also their intracellular fate. Similarly to other nanomaterials based on carbon for multi-targeted therapies and imaging [9,10], CNTs functional nanomaterials were endowed with unique properties generated by the synergic actions of components [3].

Non-covalent modification of CNTs with porphyrinoids is a well-investigated strategy to modulate the environment of chromophores, thus improving the light absorption and emission features [11], charge-transport [12] or energy transfer properties [13] in view of bio-labeling and light harvesting applications. In particular, it is well-known as π-stacking of CNTs with hydrosoluble porphyrins provides donor-acceptor complexes with efficient energy transfer [14]. Porphyrin free bases or metallo porphyrins/DNA supramolecular systems undergo strong charge transfer with semiconducting CNTs [15]. Enhanced photoconductivity has been reported for J- and H- porphyrin aggregates (head-to-tail or head-to-head molecules stacking, respectively) obtained in solution by interaction with single-walled CNTs (SWCNTs) [16] or at solid state with double-walled CNTs (DWCNTs) [17] or by decorating MWCNTs film [18]. Moreover, recently it was demonstrated that photoluminescence properties of a hybrid material assembled by formation of J-aggregates of benzo[e]indocarbocyanine (BIC) on SWCNTs can be modulated by selecting cis- or trans- isomer of the dye: the first one quenches the photoluminescence by strong interaction with CNTs, whereas the second one forms free J-aggregates characterized by photoluminescence bands of practical use in biomedical imaging [19]. Indeed, it is well known that J-aggregates feature very narrow red-shifted absorption bands, showing renewed optical, photophysical, and structural properties vs. monomer [20]. In this framework, multifunctional nanotheranostic based on J-type aggregates of cyanine [21,22], bacterio-pheophorbide [23], chlorine [24], and Bodipy [25] were proposed due to their excellent photothermal and/or NIR absorbing features for applications in imaging guided therapy (i.e., photoacustic imaging). However, these J-type aggregates generally need to be entrapped in liposomes or dispersed in surfactants to increase their solubility and bio-availability.

Within the incoming research of composite nanomaterials, our interest has been addressed to J-aggregates of meso-tetrakis-(4-sulfonatophenyl)porphyrin (TPPS) exhibiting peculiar optical features [26,27,28,29]. Such features can be fine-tuned depending on the strategy adopted to obtain the structure, i.e., by selecting appropriate polyamines as scaffolds [30,31,32,33,34,35] or by tailoring nanomaterials (i.e., metal nanoparticles) [36,37,38,39,40] or triggering porphyrin J-aggregation by modulating pH and/or ionic strength [41,42,43,44]. TPPS J-aggregates, in line of principle, would not necessitate further manipulation/encapsulation to explicate their properties within cells or tissues. These aggregated species could lead to stimuli-responsive therapeutic action upon irradiation on their extinction bands, fluorescence probing in cellular environments, or refilling of the dye upon eventual J-aggregates disassembly [45,46] in biological sites [47].

Regarding the design of SWCNTs/TPPS nanohybrids, assembly between TPPS with amine-conjugated SWCNTs has been obtained in water, pointing out that the photophysical properties of porphyrin are largely influenced by length of CNTs amine chain [48] whereas TPPS J-aggregates on SWCNTs have been prepared in organic solvents [49].

Herein, we report on the formation of relatively stable TPPS J-aggregates wrapped to covalently amine-modified MWCNTs in aqueous solution. We anticipate that, in the presence of tetraethylenepentamine-functionalized MWCNTs (MWCNT-EPA) in mild acidic conditions, TPPS porphyrin easily self-assembles into J-aggregates exhibiting peculiar extinction bands in the visible region (i.e., ≅493 nm) and in the therapeutic window (i.e., ≅710 nm), together with an emission band in the red spectral region for potential phototherapeutic and/or photodiagnostic applications. Conversely, ethylenediamine-modified MWCNTs (MWCNT-EDA) do not induce J-aggregate formation due to EDA structural features vs. EPA ones. The MWCNT-EPA/TPPS J-aggregates are stable in mimicking physiological medium (NaCl = 0.15 M), thus opening the route for their potential application in dual therapeutic/diagnostic assessment. 

## 2. Materials and Methods 

The 5,10,15,20-tetrakis(4 sulfonatophenyl)porphyrin (TPPS), MWCNTs, tert-butyl 2-aminoethylcarbamate (EDABoc), tetraethylenepentamine (EPA), N-(3-dimethylaminopropyl)-N′-ethylcarbodiimide hydrochloride (EDC), 1-Hydroxybenzotriazole (HOBt), Ninhydrin test kit, other solvents and reagents were purchased from Sigma-Aldrich Chemicals (Milan, Italy). The stock porphyrin aqueous solutions were freshly prepared and their concentrations were determined using the extinction coefficient at the Soret maximum (ε = 5.33 × 10^5^ M^−1^cm^−1^ at λ = 414 nm). All the reagents were used without further purification and all the solutions were prepared in dust free Milli-Q water (Merck, Darmstadt, Germany). 

Carboxylated multiwalled carbon nanotubes (MWCNT-Ox) were prepared by the oxidation of MWCNTs (mean diameter 5–10 nm; average length 10–20 µm) with sulfuric acid/nitric acid (3:1 *v*/*v*, 98% and 69%) according to the protocol previously reported [50]. MWCNT-Ox (100 mg) in 25 mL of dry DMF were sonicated for 30 min, then EDC (112 mg, 0.58 mmol) and HOBt (40 mg, 0.30 mmol), were added and the black suspension was stirred at room temperature for one hour. EDABoc (55 mg, 0.24 mmol) was added and the reaction was stirred for 72 hours. Water/ethanol (1:1 mixture) was added and the crude reaction mixture was filtered under vacuum (Millipore, 0.1 µm), washed with an excess of water/ethanol and finally diethyl ether. The resulting MWCNT-EDABoc were dispersed in dioxane (10 mL) and treated with 5 mL of HCl 4 M at room temperature for 4 h. The mixture was filtered under vacuum (Millipore, 0.1 µm) and the precipitate was treated with 5 mL of triethylamine/water (1:4), thus obtaining MWCNT-EDA. This was washed several times with water/ethanol by successive bath sonication and centrifugation (8000 rpm 10 min) procedures and finally dried at 60 °C to give 60 mg of material. The amount of free amine groups on MWCNT-EDA was estimated by Kaiser test (0.22 mmol/g). 

MWCNT-EPA were prepared by the coupling of MWCNT-Ox and EPA, in presence of EDC/HOBt, according to the amidation reaction procedure above described. The resulting MWCNT-EPA were washed several times with water/ethanol by successive bath sonication and centrifugation (8000 rpm 10 min) procedures and finally dried at 60 °C. Termogravimetric analysis (TGA) data indicated a weight loss of about 4.3% at 500 °C, which roughly corresponds to 0.22 mmol/g of EPA. The ninhydrin assay indicated an amount of free amino groups of 0.44 mmol/g.

Primary amine loadings were measured spectroscopically using the colorimetric Kaiser conditions [51,52]. Commercial Kaiser test kit is composed of three solutions as it follows: (a) 0.5 g/mL of phenol in absolute EtOH; (b) 2 mL of potassium cyanide 1 mM (aqueous solution) dissolved in 98 mL of pyridine; (c) 0.05 g/mL of ninhydrin in absolute EtOH. Briefly, 0.5 mg of MWCNT-EDA or MWCNT-EPA were treated in sequence with 75 μL of solution (a), 100 μL of solution (b) and 75 μL of solution (c). The dispersion was sonicated in a water bath and then was heated at 120 °C for 5 min, diluted with 4750 μL of absolute EtOH and centrifugated at 14,000 rpm. The absorbance at 570 nm of supernatant was correlated to the amount of free amine groups on MWCNTs surface (NH_2_ loading (mmol/g)); using the following equation:

(1) [free amines]=([Abs] × dilution × 1000)/(ε × sample weight  × optical path)

where dilution was fixed to 5 mL, optical path was 1 cm; sample weight was 0.5 mg; extinction coefficient (ε) was 15,000 M^−1^ cm^−1^.

Dispersions of MWCNT-EDA and MWCNT-EPA (0.43 mg/mL) were prepared in 10 mM citrate buffer by bath sonication for 20 min. For the experiments, a volume of 100 μL has been used and diluted to a final concentration of 0.02 mg/mL. The interaction of TPPS with the two batches of MWCNTs has been investigated in citrate buffer solution (10 mM, pH 2.4) following two different mixing order procedures: (i) porphyrin-first protocol (PF) and (ii) porphyrin- last protocol (PL), consisting of the addition of a proper volume of MWCNTs dispersion to a diluted TPPS solution in citrate buffer and of the addition of TPPS from a stock solution to a diluted MWCNTs dispersion, respectively [43,53]. In some experiments MWCNT- EPA in citrate buffer has been previously mixed with NaCl (0.15 M), followed by the addition of TPPS. In all the experiments, the final concentration of TPPS was 5 µM.

UV-Vis spectra have been collected on a diode-array spectrophotometer Agilent model 8452. The circular dichroism (CD) spectra were recorded on a JASCO J-720 spectropolarimeter, equipped with a 450 W xenon lamp. CD spectra were corrected both for the cell and buffer contributions. A Jasco mod. FP-750 spectrofluorometer has been used to record fluorescence emission and Resonance Light Scattering (RLS) spectra. Emission spectra were not corrected for the absorption of the samples and a synchronous scan protocol with a right angle geometry was adopted for collecting RLS spectra [54]. All the aqueous dispersions were analysed by using a 1 cm optical path cuvette.

TGA was performed by using PerkinElmer Instruments Pyris1 TGA at a heating rate of 10 °C/min over the range from room temperature (r.t) to 1000 °C under N_2_ atmosphere.

A TEM, JEM2100 LaB, working at 100 kV, and a digital Scanning transmission electron microscopy (STEM) set with BF & DF STEM Detectors plus SE/BSE detector (University of Exeter, UK) were used to investigate morphology of MWCNTs and MWCNTs/TPPS J-aggregates. Samples were prepared by evaporating ten drops of the aqueous dispersions of the investigated system more days after mixing (1–3 days) on 300 mesh holey-carbon coated copper grids.

## 3. Results and Discussion

### Synthesis and Characterization of Amine Multiwalled Carbon Nanotubes 

**Figure 1:**
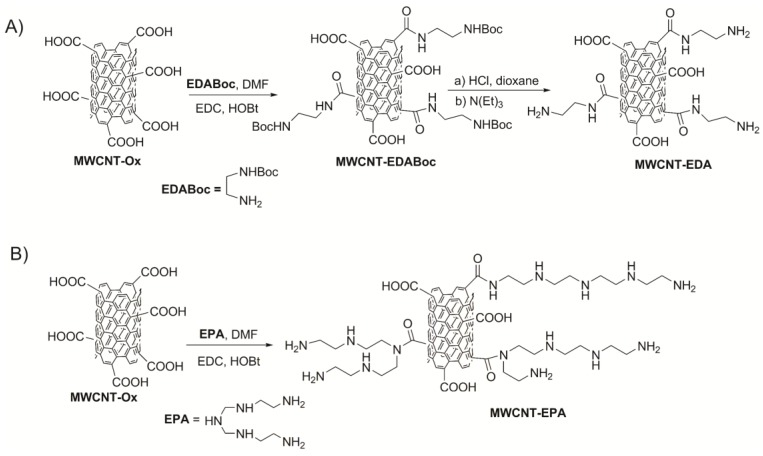
Schematic representations of MWCNT-EDA (**A**) and MWCNT-EPA (**B**) synthetic procedures.

Amine multiwalled carbon nanotubes, MWCNT-EDA and MWCNT-EPA, were prepared by coupling of carboxylated MWCNTs (MWCNT-Ox) with tert-butyl 2-aminoethylcarbamate (EDABoc) or tetraethylenepentamine using EDC/HOBt in DMF according to Figure 1A,B, respectively. MWCNT-Ox were prepared by the oxidation (HNO_3_/H_2_SO_4_ 1:3, 6 h, 60 °C) of commercially available multiwalled carbon nanotubes according to a previously reported procedure [8,50]. 

**Figure 2:**
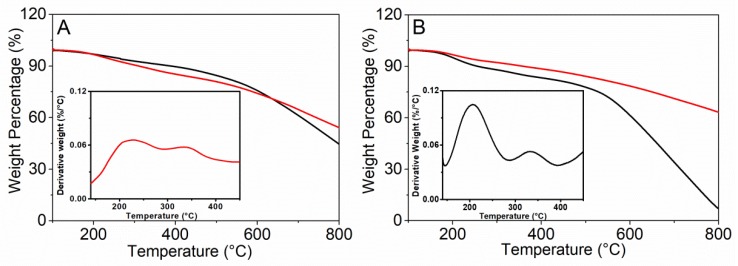
TGA profiles of MWCNT-Ox (dark line) and MWCNT-EPA (red line) (**A**). TGA profiles of MWCNT-EDABoc (dark line) and MWCNT-EDA (red line) (**B**). In the insets DTG curve of MWCNT-EPA (A) and MWCNT-EDABoc (B). TGA analyses were carried out under N_2_ atmosphere.

The degree of functionalization of MWCNTs was investigated by TGA analysis (Figure 2) and the primary amines loadings was determined using the colorimetric Kaiser test [51,52].

The TGA curve of MWCNT-Ox shows a gradual weight loss of about 15.5% at 500 °C (Figure 2A). MWCNT-EPA TGA profile displays two weight loss steps in the range 100–400 °C, likely due to decomposition of polyamine alkyl chain (inset of Figure 2A). From TGA data, a weight loss of about 4.3% at 500 °C which roughly corresponds to 0.22 mmol/g of EPA (Figure 2A) has been estimated. By the correlation with the absorbance at 570 nm using the ninhydrin assay, an amount of free amino groups of 0.44 mmol/g has been determinated, probably suggesting a role of secondary amine groups in the amidation reactions (Figure 1B). 

According to literature data, a higher thermal stability of MWCNTs containing free amine groups has been detected with respect to the MWCNTs sample containing Boc-amine groups (MWCNT-EDA vs. MWCNT-EDABoc, Figure 2B) [55,56]. The significant weight loss of MWCNT-EDABoc in the range 100–300 °C (see DTG, inset Figure 2B) can be attributed to the thermal decomposition and rearrangement of the tert-butoxyl groups. On the basis of TGA data, it was not realistic to determine the degree of functionalization in terms of weight loss (∆m ≈ 0.9–1%) [57]. Thus, we have estimated the amount of free amine functional groups by the Kaiser test (0.22 mmol/g).

**Figure 3:**
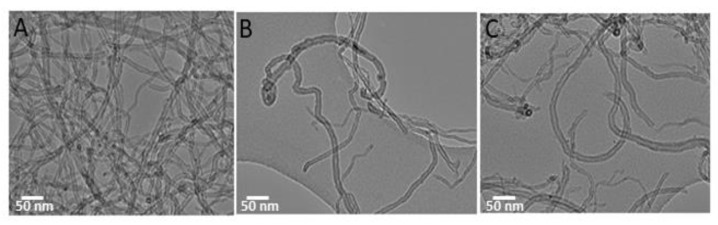
TEM images of MWCNT-Ox (**A**), MWCNT- EDA (**B**) and MWCNT-EPA (**C**).

TEM analyses of functionalized MWCNTs indicated that the chemical functionalization with amine groups preserved the characteristic morphology of multiwalled tubes scaffold. An average external diameter of ~10 nm, corresponding to an average number of 8–10 layers were found. MWCNT-ox appeared strongly aggregates in bundles (Figure 3A), whereas well distinct isolated MWCNTs are observed in TEM image of amine functionalized MWCNTs (MWCNT-EDA, Figure 3B and MWCNT-EPA, Figure 3C). Moreover, all the functionalized MWCNTs (MWCNT-Ox, MWCNT-EPA and MWCNT-EDA) were shortened by oxidation: the length was reduced from the micrometre (pristine MWCNTs, see Supplementary Figure S1) to nanometre scale [8,50].

**Figure 4:**
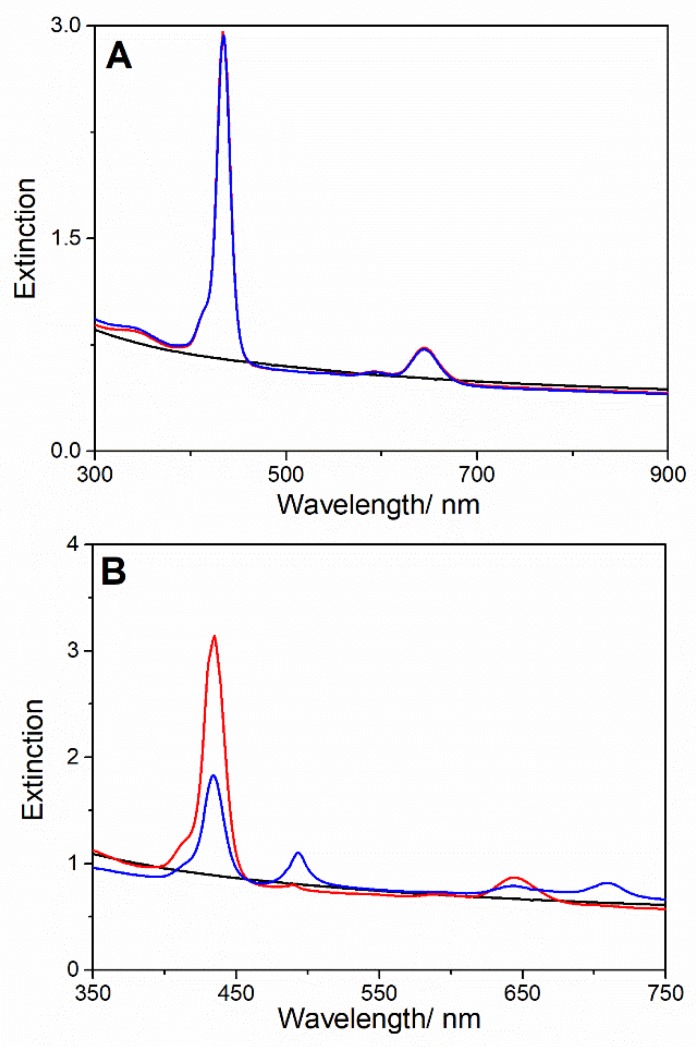
UV-Vis spectra of MWCNT-EDA (black line) and upon TPPS addition (red line), and 1 day after mixing (blue line) (**A**), and of MWCNT-EPA (black line), upon TPPS addition (red line), and 1 day after mixing (blue line) (**B**). Experimental conditions: [TPPS] = 5 µM; MWCNT-EDA or MWCNT-EPA = 0.02 mg/mL; 10 mM citrate buffer at pH 2.4; PL protocol; T = 298 K.

In order to obtain MWCNT-EDA/TPPS J-aggregates hybrids, the two aforementioned mixing order protocols (both PF and PL) have been used under mild acidic condition. Whatever of the procedure employed, we found that the extinction features of free diacid porphyrin (B band centered at 434 nm and Q bands at 592 and 645 nm) remained unchanged even after 1 day (Figure 4A). No TPPS J aggregates were formed and no interaction between TPPS and MWCNT-EDA was revealed. This behavior could be ascribable to the presence of only one pendant amino group for chain in EDA, and this observation agrees with previous results on the role of the amine chain length in inducing the aggregation of TPPS [53]. 

On the other hand, upon the addition of the porphyrin to the MWCNT-EPA dispersion, the UV-Vis profile shows the spectral signatures both of the diacid form of TPPS (B-band at 434 nm) and of J-aggregates evidenced by their typical extinction band arising at 489 nm. During the time, we observed the decrease of the intensity of the diacid band accompanied by the increase of the intensity of J-band, which furthermore undergoes a bathochromic shift from 489 to 493 nm. After one day, at the end of the aggregation process, UV-Vis spectrum of MWCNT-EPA/TPPS exhibits the B and Q bands ascribable to the residual monomeric diacid form of TPPS and J-aggregates (Figure 4B). It is noteworthy that the formation of J-aggregates does not occur under the same experimental conditions in absence of MWCNT-EPA, but it can be forced by decreasing the pH of the medium [42]. On the bases of the experimental evidences, we suggest that only EPA functionalized MWCNTs are able to trigger the TPPS aggregation process. This could be ascribable to the occurrence of an initial electrostatic interaction among a sufficient number of positively charged protonated amino groups on the CNTs surface and negatively charged sulfonated groups present in the periphery of the dyes [31,58]. Moreover, the observed red-shift of the extinction B-band of J-aggregates in the time could suggest a rearrangement of the aggregates due to an interaction with different amine-modified carbon nanotubes or their location in a different microenvironment with respect to the aqueous solution.

**Figure 5:**
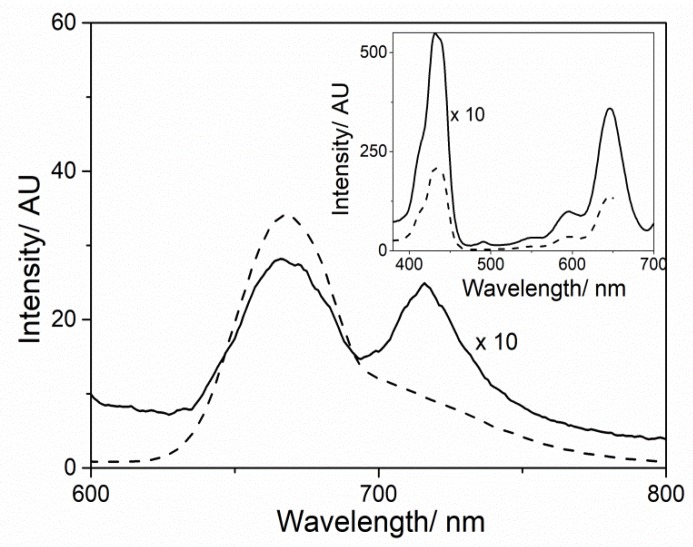
Fluorescence emission spectra of MWCNT-EPA/TPPS J-aggregates system 1 day after mixing (dashed and solid lines recorded at λ_exc_ = 455 and 493 nm, respectively) and, in the inset, the corresponding excitation spectra (dashed and solid lines recorded at λ_em_ = 669 and 717 nm, respectively). Experimental conditions: [TPPS] = 5 µM; MWCNT-EPA = 0.02 mg/mL; 10 mM citrate buffer at pH 2.4; PL protocol; T = 298 K.

The emission spectrum (λ_exc_ 455 nm), at the end of the aggregation process, shows the typical fluorescence emission of the diacid form of TPPS centered at 669 nm. Further, upon excitation on the J-aggregates band (λ_exc_ 493 nm), an emission band at 717 nm can be also detectable (Figure 5). The different ratio of the bands intensity at 669 and 717 nm at the two distinctive excitation wavelengths is due to the fluorescence emission generated from the aggregated species. This evidence is confirmed by the related excitation spectra (Figure 5 inset) showing spectral features for both the diacidic TPPS and J-aggregates. However, the monomeric porphyrin is the predominant species in the excitation profile at both emission wavelengths, due to its longer lifetime value with respect to the J-aggregate one [59,60].

**Figure 6:**
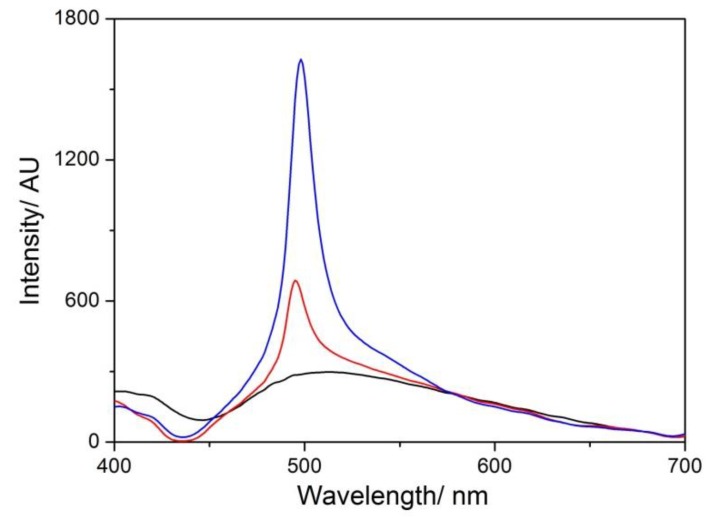
RLS spectra of MWCNT-EPA (black line), after TPPS addition (red line), and 1 day after mixing (blue line). Experimental conditions: [TPPS] = 5 µM; MWCNT-EPA = 0.02 mg/mL; 10 mM citrate buffer at pH 2.4; PL protocol; T = 298 K.

The RLS spectra recorded soon after mixing shows a very sharp peak in the red region of the extinction band which increases in intensity at the end of the aggregation process. These findings agree with our previous results [42], pointing to the formation of self-assemblies of electronically coupled porphyrins, which cause a large enhancement of the resonant light scattering [54] at the red-edge of the extinction peak (Figure 6).

J-aggregates of TPPS induced by MWCNT-EPA were stable in acidic aqueous dispersion for a day or more after preparation. In difference with our previous findings on polyamine-mediated J-aggregates [30,31,61], the optical profiles are in line with the usual Frenkel exciton theory, rather than in terms of an extended network formed by the J-aggregates and amine modified MWCNTs in which dipole–dipole coupling among single porphyrins takes place. 

**Figure 7:**
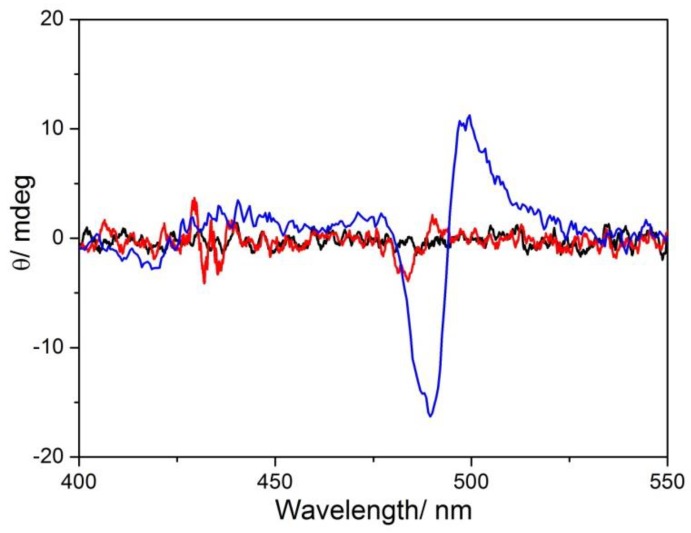
CD spectra of MWCNT-EPA (black line), soon after TPPS addition (red line), and 1 day after mixing (blue line). Experimental conditions: [TPPS] = 5 µM; MWCNT-EPA = 0.02 mg/mL; 10 mM citrate buffer at pH 2.4; PL protocol; T = 298 K.

CD spectra were recorded after freshly mixing of the components and at the end of the aggregation process (Figure 7). As expected for achiral MWCNT-EPA, CD spectrum is silent before the addition of the chromophoric species, so confirming the absence of optical activity for amine modified CNTs. On the other hand, when TPPS was added a slight bisegnate positive Cotton effect in the aggregates absorption region has been observed. At the end of the aggregation process, an increase in intensity and a red shift of the CD profile were observed. This behavior, observed by means of spectroscopic and light scattering techniques, is due to the formation of large and rearranged structures as the result of the interactions among porphyrin aggregates and functionalized MWCNTs. 

**Figure 8:**
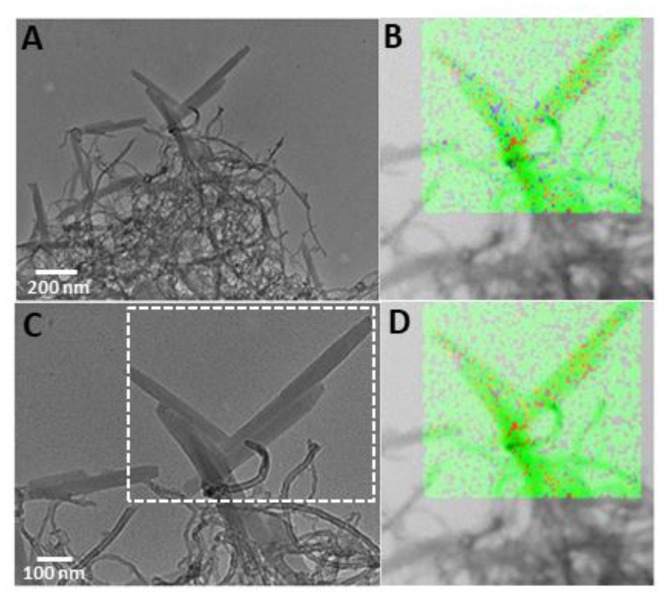
TEM images of MWCNT-EPA/TPPS J-aggregates at low (**A**) and high resolution (**C**): (**B**,**D**) corresponds to STEM analysis of total (C/N/O/S) and C/S merging of elements distribution respectively taken within the dashed region of the assemblies in (**C**) (for individuals colours of elements refers to (**B**) in Figure S3; see Materials and Methods for preparation conditions).

In agreement with the spectroscopic characterization, representative TEM images of MWCNT-EPA/TPPS J-aggregates pointed to the coexistence of both MWCNT-EPA and TPPS J-aggregates in the same area (Figure 8 and Supplementary Figure S2). In particular, TPPS J-aggregates with an average diameter of 45 nm and length of 250–500 nm seem to be wrapped by separate nanotubes or bundles of MWCNT-EPA having an average external diameter of about 15 nm. 

STEM analysis of MWCNT-EPA/TPPS J-aggregates shows the total elemental distribution pointing out the presence of carbon and oxygen for MWCNT-EPA, and carbon, oxygen, sulfur and nitrogen for TPPS J-aggregates (Supplementary Figure S3). Interestingly, in the marked area (Figure 8C), the total elements merging (Figure 8B) appears to be similar to the carbon/sulfur merging (Figure 8D). These results evidence the co-localization of TPPS J-aggregates and carbon nanotubes in the investigated samples. Since self-organization phenomenon is a hierarchical process, it is well known as the morphology of final aggregates can be controlled by the mixing order protocol [43]. In this framework, we performed experiments by adding amine carbon nanotubes to diacid TPPS (PF protocol). Surprisingly, at the end of the aggregation process, all the spectroscopic (Supplementary Figures S4 and S5) and morphological features (Supplementary Figure S6) show no change with respect to that observed by previous reagent mixing order protocol (PL). Because, in the case of the self-aggregation of neat TPPS in acidic conditions [43], the mixing order protocol is strictly related to the occurrence of porphyrin nucleation phenomena, here, we are prone to think that MWCNT-EPA could act as nucleation centers, inducing dye aggregation independently by the mixing order protocol. 

**Figure 9:**
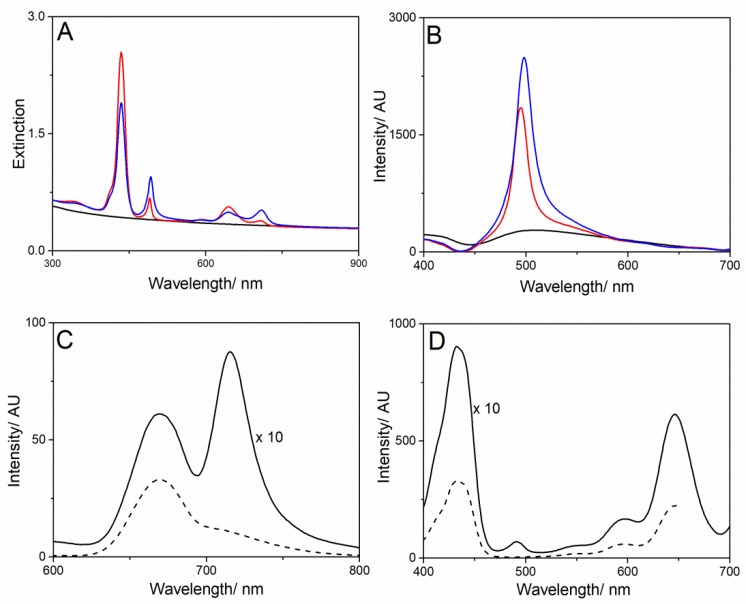
UV- Vis spectra (**A**) and RLS ( **B**) of MWCNT-EPA (black line), upon TPPS addition (red line) and 4 h after mixing (blue line); (**C**) Fluorescence emission spectra of MWCNT-EPA/TPPS J-aggregates (dashed and solid lines at λ_exc_ = 455 and 492 nm, respectively) and (**D**) the corresponding excitation spectrum ( dashed and solid lines at λ_em_ = 669 and and 717 nm, respectively). Experimental conditions: [TPPS] = 5 µM; MWCNT-EPA = 0.02 mg/mL; 10 mM citrate buffer at pH 2.4; [NaCl] = 0.15 M, PL protocol; T = 298 K.

In order to verify the stability of MWCNT-EPA/TPPS J-aggregates in mimicking physiological medium (NaCl 0.9% *w*/*w* ≅ 0.15 M), the system has been prepared by firstly dispersing MWCNT-EPA in NaCl 0.15 M aqueous solution and then adding TPPS. Under these conditions, the spectroscopic evidences of the final system remain almost unchanged (Figure 9) with respect to the unsalted solutions thus confirming the formation of chromophoric assemblies. The premixing of MWCNTs and NaCl, followed by the addition of porphyrin, seems to lead to a larger amount of J-aggregates due to the ionic strength effect [62]. Generally, optical stability for J-aggregates is difficult to achieve. Therefore, the use of surfactans or the entrapment in liposomes of the dye forming J-aggregates were experienced in literature [21]. In our case, MWCNTs induce, whatever the preparation procedure, the formation of stable J-aggregates able to retain their optical properties even after more days (1–3 days). Therefore, no further manipulation to preserve their pristine optical properties was necessary. 

Altogether, the combination of drug carrier ability of MWCNTs with the theranostic properties of porphyrins could allow the development of MWCNTs/TPPS J-aggregates nanohybrids for applications in biomedical field. Unlike from the others families of hybrid carbon nanomaterials [9], the potential applications in biological/pharmaceutical field of carbon based nanomaterials- porphyrins appear still scarcely investigated, especially for J-aggregates self-assemblies as well as their intracellular trafficking, therapeutic and imaging properties. In the literature, it was observed that hybrids nanomaterials based on porphyrinoids [24,63] were prepared at pH different from physiological conditions, and then treated with cells. In this context, future work will be devoted to studying the biocompatibility and cellular uptake of our hybrid MWCNTs/J-aggregates supramolecular systems. With these perspectives in mind, this research could lay the groundwork for the incoming biological assessment of MWCNT-EPA/TPPS J-aggregates. 

## 4. Conclusions

MWCNTs can be easily functionalized by covalently introducing pendant amino-groups on their surface. In this paper we used ethylenediamine (EDA) and tetraethylenepentamine (EPA), which after coupling with carboxylic groups on the exterior walls, led to one and four protonable amino groups for chain, respectively. Under mild acidic conditions, the diacid form of TPPS is able to electrostatically bind to the surface and eventually aggregate. In line with our previous investigations on the ability of polyamines to trigger the aggregation of TPPS, the presence of only one pendant amino group (EDA) is not enough to induce the formation of TPPS J-aggregates, whatever the mixing protocol. On the other hand, when EPA functionality is present, these species are effective to generate stable MWCNT-EPA/TPPS J-aggregates nanohybrids and their general spectroscopic features are rather independent on the mixing protocol. A similar behavior was observed in solutions mimicking physiological medium (NaCl ≅ 0.15 M), whereby stable nanohybrids were also obtained. These systems exhibit remarkable optical features, and in this perspective could be considered for potential applications in phototherapy (by irradiating on extinction bands at 491 nm and/or at 709 nm) and/or bio-imaging (by exploiting the fluorescent emission band at 716 nm). In this respect, this class of amine-modified MWCNTs could be investigated as carriers of J-aggregates in biological environment. All these optical and structural properties make MWCNT-EPA/TPPS J-aggregates appealing for further considerations in theranostic.

## Supplementary Materials

The following are available online at https://www.mdpi.com/2079-4991/10/4/669/s1, Figure S1: TEM image of pristine MWCNTs; Figure S2: TEM images of MWCNT-EPA/TPPS J-aggregates; Figure S3: STEM of MWCNT-EPA/TPPS J-aggregates; Figure S4: UV-Vis spectra of an aqueous solution of TPPS J-aggregates/MWCNT-EPA (PF protocol) and MWCNT-EPA TPPS J-aggregates (PL protocol); Figure S5: CD spectra of an aqueous solution of TPPS J-aggregates/MWCNT-EPA (PF protocol) and MWCNT-EPA/TPPS J-aggregates (PL protocol); Figure S6: TEM images of TPPS J-aggregates/MWCNT-EPA (PF protocol).
