# Supplementary material for: Novel Nanohybrids Based on Supramolecular Assemblies of Meso-tetrakis-(4-sulfonatophenyl) Porphyrin J-aggregates and Amine-Functionalized Carbon Nanotubes
Source: Nanomaterials (Basel). 2020 Apr 2;10(4):669. doi: 10.3390/nano10040669 (PMC7221796; doi:10.3390/nano10040669)
Supplement: Supplementary file 1 [file nanomaterials-10-00669-s001.pdf]

Electronic Supporting Information:

Novel Nanohybrids based on Supramolecular Assemblies of Meso-tetrakis-(4-sulfonatophenyl) Porphyrin J-aggregates and Amine- functionalized Carbon Nanotubes

Mariachiara Trapani [1], Antonino Mazzaglia [1,*], Anna Piperno [2,3], Annalaura Cordaro [1, 2], Roberto Zagami [1], Maria Angela Castriciano [1*], Andrea Romeo [1, 2, 4] and Luigi Monsù Scolaro [1, 2, 4]

[1] CNR-ISMN, Istituto per lo Studio dei Materiali Nanostrutturati c/o Dipartimento di Scienze Chimiche, Biologiche, Farmaceutiche ed Ambientali, Università of Messina, V. le F. Stagno D'Alcontres 31, 98166 Messina, Italy

[2] Dipartimento di Scienze Chimiche, Biologiche, Farmaceutiche ed Ambientali , Università of Messina, V. le F. Stagno D'Alcontres 31, 98166 Messina, Italy

[3] Consorzio Interuniversitario Nazionale di Ricerca in Metodologie e Processi Innovativi di Sintesi, C.I.N.M.P.I.S., Unità Operativa dell'Università di Messina V. le F. Stagno D'Alcontres, 3198166 Messina, Italy

[4] Consorzio Interuniversitario di Ricerca in Chimica dei Metalli nei Sistemi Biologici, C.I.R.C.M.S.B, Unità Operativa dell'Università di Messina, V. le F. Stagno D'Alcontres, 31, 98166 Messina, Italy

* Correspondence: antonino.mazzaglia@cnr.it (A.M.); maria.castriciano@cnr.it (M.A.C.)

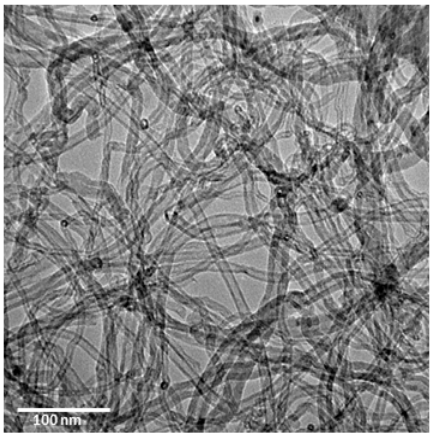

Figure S1 TEM image of pristine MWCNTs (commercially available).

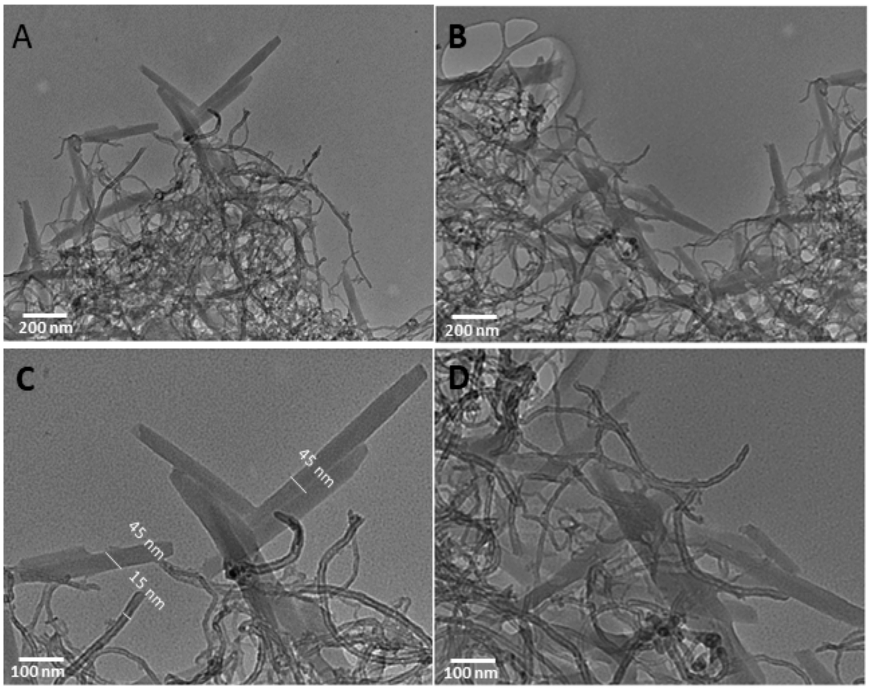

Figure S2 TEM images of MWCNT-EPA/TPPS J-aggregates at low (**A**) and (**B**), and at high resolution (**C**) and (**D**) of two different parts of the same sample (A corresponds to C and B to D; PL protocol: see Materials and Methods for preparation conditions).

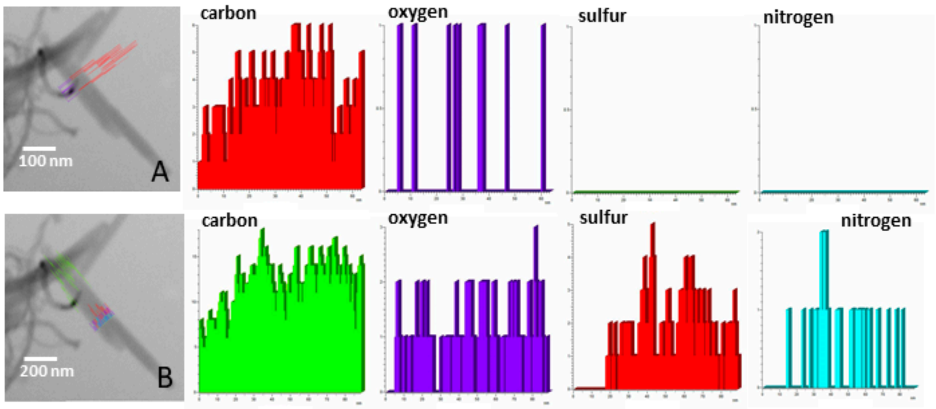

Figure S3 STEM of MWCNT-EPA/TPPS J-aggregates with linescans displaying total elemental analysis taken at 65 nm scale length. Elemental analysis was detected by peculiar element emission lines for MWCNT-EPA (**A**) and for TPPS/J-aggregates (**B**).

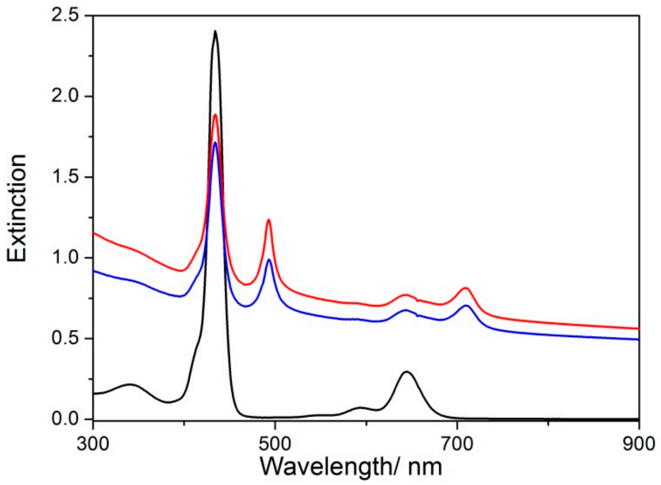

Figure S4 UV-Vis spectra of an aqueous solution of TPPS (black line) and after MWCNT-EPA addition (red line, PF protocol). As comparison the spectrum of MWCNT-EPA/TPPS J-aggregates system prepared using PL protocol is reported (blue line). Experimental conditions: [TPPS] = 5 μM; MWCNT-EPA= 0.02 mg/mL; 10 mM citrate buffer at pH 2.4; aging time: 1 day, T= 298 K.

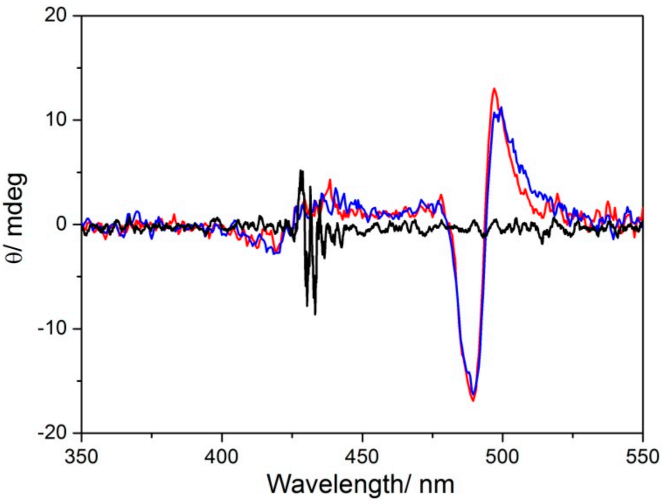

Figure S5 CD spectra of an aqueous solution of TPPS (black line) and after MWCNT-EPA addition (red line, PF protocol). As comparison the spectrum of MWCNT-EPA/TPPS J-aggregates system prepared using PL protocol is reported (blue line). Experimental conditions: [TPPS] = 5 μM; MWCNT-EPA= 0.02 mg/mL; 10 mM citrate buffer at pH 2.4; aging time: 1 day, T= 298 K.

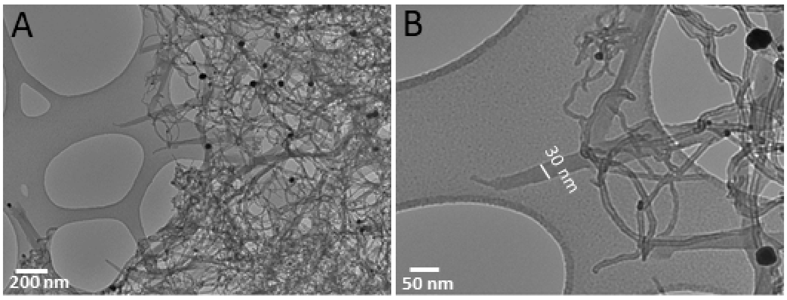

Figure S6 TEM images of TPPS J-aggregates/ MWCNT-EPA at low (**A**) and high resolution (**B**) (PF protocol: see Materials and Methods for preparation conditions).
